# Plant-Based Foods and Their Bioactive Compounds on Fatty Liver Disease: Effects, Mechanisms, and Clinical Application

**DOI:** 10.1155/2021/6621644

**Published:** 2021-03-01

**Authors:** Hang-Yu Li, Ren-You Gan, Ao Shang, Qian-Qian Mao, Quan-Cai Sun, Ding-Tao Wu, Fang Geng, Xiao-Qin He, Hua-Bin Li

**Affiliations:** ^1^Guangdong Provincial Key Laboratory of Food, Nutrition and Health, Department of Nutrition, School of Public Health, Sun Yat-Sen University, Guangzhou 510080, China; ^2^Research Center for Plants and Human Health, Institute of Urban Agriculture, Chinese Academy of Agricultural Sciences, Chengdu 610213, China; ^3^School of Food and Biological Engineering, Jiangsu University, Zhenjiang 212001, China; ^4^Institute of Food Processing and Safety, College of Food Science, Sichuan Agricultural University, Ya'an, China; ^5^Key Laboratory of Coarse Cereal Processing (Ministry of Agriculture and Rural Affairs), School of Food and Biological Engineering, Chengdu University, Chengdu, China

## Abstract

Fatty liver disease (FLD), including nonalcoholic fatty liver disease (NAFLD) and alcoholic fatty liver disease (AFLD), is a serious chronic metabolic disease that affects a wide range of people. Lipid accumulation accompanied by oxidative stress and inflammation in the liver is the most important pathogenesis of FLD. The plant-based, high-fiber, and low-fat diet has been recommended to manage FLD for a long time. This review discusses the current state of the art into the effects, mechanisms, and clinical application of plant-based foods in NAFLD and AFLD, with highlighting related molecular mechanisms. Epidemiological evidence revealed that the consumption of several plant-based foods was beneficial to alleviating FLD. Further experimental studies found out that fruits, spices, teas, coffee, and other plants, as well as their bioactive compounds, such as resveratrol, anthocyanin, curcumin, and tea polyphenols, could alleviate FLD by ameliorating hepatic steatosis, oxidative stress, inflammation, gut dysbiosis, and apoptosis, as well as regulating autophagy and ethanol metabolism. More importantly, clinical trials confirmed the beneficial effects of plant-based foods on patients with fatty liver. However, several issues need to be further studied especially the safety and effective doses of plant-based foods and their bioactive compounds. Overall, certain plant-based foods are promising natural sources of bioactive compounds to prevent and alleviate fatty liver disease.

## 1. Introduction

Fatty liver disease (FLD) has become one of the most common chronic diseases in the world, and there are two major types of FLD, including nonalcoholic fatty liver disease (NAFLD) and alcoholic fatty liver disease (AFLD) [1]. NAFLD affects approximately 1.7 billion individuals worldwide, which is almost equivalent to 25% of the global population [2, 3]. In China, the prevalence of NAFLD has been dramatically increased from 18% to 29% in recent decades [4]. Similarly, the prevalence of NAFLD has increased by 11% in men and 3% in women in Korea over the past 19 years [5]. On the other hand, although the abstinence of alcohol intake is the most effective way to prevent AFLD, the truth is, from 2001 to 2016, the total prevalence of alcohol-induced fatty liver increased from 4.3% to 4.7% in the USA [6]. Moreover, a cross-sectional study reported that the prevalence of AFLD in male drinkers has increased from 22.3% to 36.6% in Japan over the last two decades [7]. In short, FLD is emerging as the priority of global health issues and intimately associated with an unhealthy lifestyle.

Both NAFLD and AFLD share the same fundamentally pathological characteristic, which is lipid accumulation accompanied by oxidative stress and inflammation in the liver, but the inducers are different [8]. NAFLD is commonly induced by unhealthy dietary patterns, such as a high-fat diet, over intake of fructose, and animal protein [9]. On the other hand, chronic excessive consumption of alcohol is the major trigger of AFLD [10]. Steatosis in hepatocytes was closely associated with oxidative stress, inflammation, gut dysbiosis, autophagy, and apoptosis [11–13]. All these pathological mechanisms were involved in the progression of hepatic steatosis to fibrosis, cirrhosis, and even liver cancer [14]. Further studies revealed that the disorders of several important signaling pathways played a vital role in the progression of FLD, such as adenosine 5′-monophosphate-activated protein kinase (AMPK), sirtuin 1 (SIRT1), peroxisome proliferator-activated receptor (PPAR), nuclear factor erythroid 2-related factor 2 (Nrf2), toll-like receptor 4 (TLR4), and nuclear factor kappa-B (NF-*κ*B) [15–17].

The sale and consumption of plant-based food have been increased worldwide during the past two decades [18–20]. Currently, the world's market of food industry focused on how to provide more high-quality plant-based convenience foods [21]. A shift of the dietary pattern toward more plant-based food consumption is beneficial to public health [22]. Some epidemiological studies revealed that the plant-based, high-fiber, and low-fat diet was positively related to the alleviation of FLD [23–26]. Further experimental studies demonstrated that plant-based foods (such as fruits, spices, teas, and coffee) and their bioactive compounds possessed excellent properties of anti-steatosis, anti-oxidative stress, anti-inflammation, and anti-gut dysbiosis, which were related to the alleviation of FLD [27, 28]. More importantly, researchers conducted some well-designed clinical trials to better demonstrate the biological effects of plant-based food consumption in patients with FLD [29, 30]. Since the importance of such plants and bioactive compounds in preventing the prevalence of FLD, the safety assessment and the application issue in patients are worth discussing [31, 32].

To provide an updated understanding of the biological effects of plant-based foods on fatty liver diseases, this review summarized and discussed recent results from epidemiological studies, experimental studies, and clinical trials, with the emphasis focusing on their molecular mechanisms and safety assessment.

## 2. Epidemiological Evidence on the Relation Between Plant-Based Foods and FLD

Several epidemiological investigations indicated that several plant-based foods were beneficial to FLD. During 4.2 years of follow-up, a recent prospective cohort study based on 2130 NAFLD patients showed that higher consumption of fruits and vegetables decreased the risk of NAFLD in women (relative risk (RR): 0.74; 95% confidence interval (CI): 0.59, 0.93) and in men (RR: 0.75; 95%CI: 0.62, 0.92) [33]. A prospective study on 2,687 subjects showed that the higher serum level of lycopene was favorably associated with NAFLD improvement in middle-aged and elderly adults [34]. Likely, a community-based cross-sectional study involving 2,935 participants aged 40–75 years reported that lycopene was inversely relevant to NAFLD prevalence (odds ratio (OR): 0.54; 95%CI: 0.42, 0.68) [35]. Similarly, a case-control study with 196 NAFLD patients and 803 controls reported that intake of spices, like garlic and onion, could decrease the risk of NAFLD (OR: 0.36; 95%CI: 0.22, 0.56) [36]. Moreover, the OR value dropped from 1.00 to 0.71 with the increased frequency of garlic intake, suggesting that the frequency of garlic intake was inversely associated with the risk of NAFLD [37]. Besides, a multiethnic cohort study reported that the increase in coffee consumption could reduce the risks of FLD [38]. Similarly, the data from a study involving 91,436 subjects with the mean follow-up period of 2.8 years showed that the increase in coffee consumption was associated with a lower risk of FLD in Korean male [39]. In addition, several retrospectively cross-sectional studies assessed coffee intake among NAFLD patients and the result showed that the consumption of more than two cups of coffee per day could lower liver stiffness [40, 41]. Furthermore, a cross-sectional study based on 58 patients showed that the dietary pattern containing other plant-based foods like whole grains and legumes could also decrease the risk of NAFLD (OR: 0.35; 95%CI: 0.13, 0.93) [42]. Other cross-sectional studies with 1639 participants reported that the intake of whole grains could lower the risk of NAFLD (OR: 0.72; 95%CI: 0.61, 0.98) [43]. Besides, a larger cross-sectional study based on 23,529 participants showed that the consumption of insoluble dietary fiber (a type of nonstarch polysaccharide widely found in whole grains) was inversely associated with the risk of NAFLD (OR: 0.7; 95%CI: 0.61, 0.85) [44].

However, controversial results still existed among different studies. A cross-sectional study of 977 men and 1467 women from Japan showed that the association between the intake of fruit and vegetable and NAFLD was disappeared after adjustment for the body mass index [45]. What is worse, a retrospective study based on 27,214 adults showed that participants who consumed oranges over 7 times a week had a higher risk of NAFLD (OR: 1.17; 95%CI: 1.03, 1.33) [46]. This could be due to the over intake of fructose [47]. Moreover, a cross-sectional study showed that there was no statistically significant result between coffee consumption and the risk of NAFLD progression in obese individuals [48]. Also, another cross-sectional study with 2,819 middle-aged participants showed that drinking coffee was not associated with the risk of NAFLD (OR: 0.93; 95%CI: 0.72, 1.20) or AFLD (OR: 1.20; 95%CI: 0.66, 2.0) [49]. Furthermore, a cohort study examining 637 patients reported that coffee consumption was not associated with the subclinical cardiovascular diseases in NAFLD patients (hazard ratio (HR): 1.05; 95%CI: 0.91, 1.21) [50]. Additionally, the data from a two-sample Mendelian randomization analysis did not support a causal relationship between coffee intake and NAFLD risk [51].

In short, according to the results from epidemiological studies, several plant-based foods (such as garlic, onion, and whole grains), as well as their bioactive component (like insoluble dietary fiber), were beneficial to decreasing the risk of fatty liver, while excessive intake of plant-based foods containing high content of fructose (like oranges) might increase the risk of FLD. Besides, the relationship between coffee consumption and FLD needs further investigation.

## 3. Beneficial Bioactive Compounds from Plant-Based Foods in Relation to FLD

**Figure 1:**
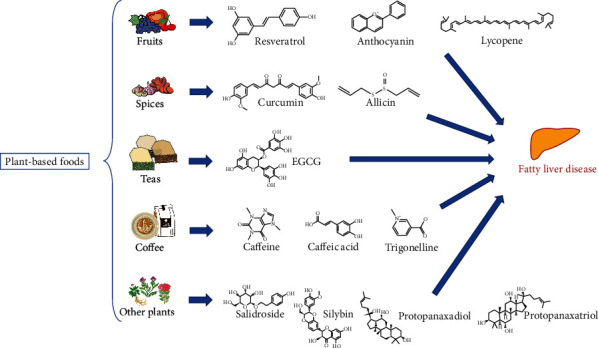
Bioactive compounds from plant-based foods relevant to FLD.

Increasing the consumption of more plant-based foods is an effective solution to tackle the severe burden of FLD. Recently, several bioactive compounds and phytochemicals (Figure 1) from plant-based foods are proven to make a significant influence on FLD, which are summarized below.

### 3.1. Beneficial Bioactive Compounds from Fruits in Relation to FLD

Resveratrol (3,5,4′-trihydroxy-trans-stilbene) is a natural phenolic stilbene widely found in grapes and possesses many beneficial properties [52]. It was formed via the condensation reaction of malonyl-CoA and 4-coumaroyl-CoA under the catalysis through the resveratrol synthase [53]. As a natural activator of SIRT1, resveratrol has been reported to prevent hepatic steatosis and even ameliorate fibrosis, and it could also improve ethanol-induced insulin resistance and regulate ethanol metabolism enzymes [54–56]. Anthocyanins are a group of water-soluble flavonoids and composed of two aromatic rings linked by three carbons in an oxygenated heterocycle, with biological effects on improving insulin resistance, liver injury, and clinical progression of NAFLD [57]. Lycopene extracted from tomato is a liposoluble carotenoid without provitamin A activity, and it could improve the status of oxidative stress and inflammation and regulate metabolic processes and liver function [58].

### 3.2. Beneficial Bioactive Compounds from Spices in Relation to FLD

Curcumin ((1*E*,6*E*)-1,7-bis(4-hydroxy-3-methoxyphenyl)-1,6-heptadiene-3,5-dione) is a natural polyphenolic compound and the major curcuminoid extracted from turmeric, consisting of two aromatic O-methoxy phenolic groups, a *β*-dicarbonyl moiety, and a seven-carbon linker containing two enone moieties [59]. Curcumin processed a variety of bioactivities such as antioxidant, anti-inflammatory, and hepatoprotective effects, and a systematic review and dose-response meta-analysis of randomized controlled trials demonstrated that curcumin could ameliorate visceral fat and abdominal obesity in NAFLD patients [60]. Allicin (diallyl thiosulfonate), a bioactive component from the aqueous extracts of garlic, could decrease the abundance of gut microbiota, like *Christensenellaceae* and *Ruminococcaceae*, in alcoholic hepatic steatosis mice, resulting in a reduction of hepatic triacylglycerol level [61, 62].

### 3.3. Beneficial Bioactive Compounds from Teas in Relation to FLD

Tea polyphenols are the bioactive compounds extracted from tea and primarily include phenolic compounds like catechins [63]. Catechins possessed several biological effects like the improvement of oxidative stress, inflammation, diabetes, and obesity, and catechins also regulated the function of metabolic tissues like the adipose tissue and liver [64]. Epigallocatechin-3-gallate (EGCG) is the most abundant catechin in teas, and it could effectively regulate energy metabolism, lipid oxidation, and insulin resistance [65, 66]. Thus, tea polyphenols could be considered a potential adjuvant therapy to modulate metabolic syndromes in FLD patients [67, 68].

### 3.4. Beneficial Bioactive Compounds from Coffee in Relation to FLD

Coffee is a popular beverage that contains a lot of bioactive compounds, such as caffeine, chlorogenic acid, cafestol, and kahweol [69]. Some bioactive compounds, like caffeic acid and trigonelline, are beneficial to protecting liver function due to their properties of improving liver triglyceride (TC) metabolism, oxidative stress, and fibrotic status; thus, these compounds are closely relevant to FLD [70].

### 3.5. Beneficial Bioactive Compounds from Other Plants in Relation to FLD

Several other plants, for example, Hongjingtian (*Rhodiola rosea*), milk thistle (*Silybum marianum*), and ginseng (*Panax ginseng*) are widely used functional foods and traditional herbs against FLD [71, 72]. Salidroside (2-(4-hydroxyphenyl)-ethyl-*β*-D-glucopyranoside) is a natural phenolic secondary metabolite from Hongjingtian (*Rhodiola rosea*), and it could improve lipid metabolism and antioxidative activity [73, 74]. Silymarin is a lipophilic active complex extracted from milk thistle (*Silybum marianum*), and it contains several flavonolignans (like silybin, silydianin, silychristin, and isosilybin), among which, silybin had the most significant hepatoprotective effects [75, 76]. Ginsenosides are amorphous compounds extracted from ginseng (*Panax ginseng*). There are two major groups of ginsenosides, the protopanaxadiol and protopanaxatriol groups, and they could suppress appetite by affecting the ventromedial hypothalamic nucleus, while inhibiting the absorption of fat in the gut [77, 78].

## 4. Mechanisms of Plant-Based Foods and Their Bioactive Compounds on FLD

The effects of plant-based foods (such as fruits, spices, teas, coffee, and other plants) and their bioactive compounds (such as resveratrol, anthocyanins, lycopene, curcumin, and tea polyphenols) on FLD have been widely investigated by *in vitro* and *in vivo* experimental studies. The potential molecular mechanisms are discussed and highlighted below.

### 4.1. Fruits and FLD

#### 4.1.1. Mechanisms of Fruits and Their Bioactive Compounds on NAFLD

Bioactive food constituents were recognized as new treatment approaches in the modulation of NAFLD. Resveratrol is widely found in plants like grapes and could attenuate NAFLD by inhibiting lipogenesis and oxidative stress in HepG2 cells [79]. Meanwhile, resveratrol is also a main compound in red wine and could improve the hepatic steatosis and redox homeostasis in HepG2 cells by upregulating the protein kinase A- (PKA-) AMPK-PPAR-*α* signaling pathway and regulating the epigenetic modification of the Nrf2 signaling pathway [80, 81]. Similarly, rutin from tomato could inhibit oxidative stress and autophagy in the NAFLD cell model by upregulating the PPAR-*α* signaling pathway, accompanied by reducing the level of TG [82]. Besides, the administration of anthocyanin extracted from sweet cherry could alleviate NAFLD in HepG2 cells and LO2 liver cells through inducing autophagy by downregulating phosphorylation of the mammalian target of rapamycin (mTOR) and protein kinase B (Akt), while upregulating the phosphorylation of AMPK [83].

In addition, *in vivo* studies also showed that the oral administration of resveratrol and anthocyanin extracted from grape improved lipid metabolism, oxidative stress, and inflammation in the NAFLD mouse model by downregulating PPAR-*γ* while upregulating Nrf2 and SIRT1 signaling pathways, accompanied by decreasing the expression of fatty acid synthase (FAS) and sterol regulatory element-binding transcription factor 1c (SREBP-1c) [81, 84]. Besides, the consumption of tomato juice could alleviate NAFLD in Sprague-Dawley (SD) rats by increasing the abundance of *Lactobacillus* and diminishing the ratio of acetate to propionate [85]. Furthermore, lycopene extracted from tomato could dose-dependently improve the liver function and lipid profiles in the NAFLD rat model by decreasing the levels of cytochrome P450 2E1 (CYP2E1), malondialdehyde (MDA), and tumor necrosis factor-*α* (TNF-*α*) in the liver [86–88]. Other fruits, like mulberry, Açai, and acerola cherry, also suppressed hepatic steatosis, oxidative stress, and inflammation in animal experiments [89–91].

**Figure 2:**
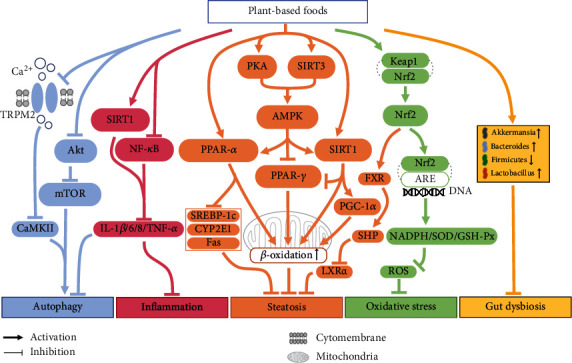
The molecular mechanisms of plant-based foods in the alleviation of NAFLD. The major molecular mechanisms include the alleviation of hepatic steatosis, oxidative stress, inflammation, gut dysbiosis, and the regulation of autophagy. To be specific, fruits, spices, and teas alleviated hepatic steatosis by activating AMPK, PPAR-*α*, SIRT1, and FXR and inhibiting PPAR-*γ* pathways. Besides, the antioxidative stress property of such plants was mainly associated with the activation of the Nrf2 pathway. Moreover, plant-based foods could alleviate inflammation by activating SIRT1, while inhibiting NF-*κ*B pathways. Furthermore, these plants alleviated gut dysbiosis by regulating the abundance of *Akkermansia*, *Bacteroides*, *Firmicutes*, and *Lactobacillus*. Also, such plants could regulate autophagy by inhibiting the Akt pathway and reducing the production of autophagic biomarkers. Akt: protein kinase B; AMPK: adenosine 5′-monophosphate-activated protein kinase; ARE: Nrf2-antioxidant response element; CYP2E1: cytochrome P450 2E1; FAS: fatty acid synthase; FXR: farnesoid X receptor; IL-1*β*/6/8: interleukin-1*β*/6/8; LXR*α*: liver X receptor *α*; mTOR: mammalian target rapamycin; NF-*κ*B: nuclear factor kappa-B; Nrf2: nuclear factor erythroid 2-related factor 2; PGC-1*α*: peroxisomal proliferator-activated receptor-gamma coactivator-1*α*; PKA: protein kinase A; PPAR-*α*/*γ*: peroxisome proliferator-activated receptor-*α*/*γ*; ROS: reactive oxygen species; SIRT1: sirtuin 1; SOD: superoxide dismutase; SREBP-1c: sterol regulatory element-binding transcription factor 1c; TNF-*α*: tumor necrosis factor-*α*.

In summary, several fruits (like grape, cherry, tomato, and mulberry) and their bioactive compounds (like resveratrol, anthocyanin, and lycopene) could be promising agents against NAFLD due to their outstanding effects on regulating lipid metabolism, oxidative stress, inflammation, and gut microbiota (Table 1), with regulating AMPK, PPAR-*α*/*γ*, Nrf2, mTOR, and Akt signaling pathways (Figure 2).

#### 4.1.2. Mechanisms of Fruits and Their Bioactive Compounds on AFLD

Several fruits and their bioactive compounds show protective effects on AFLD *in vitro*. The administration of resveratrol from fruits could alleviate AFLD in HepG2 cells by activating the AMPK-lipin1 signaling pathway, accompanied by the decreased expression of SREBP-1c and lipin1 [92]. Similarly, some natural flavonoids (like myricetin and myricitrin) extracted from berries could alleviate alcohol-induced lipid accumulation, oxidative stress, and inflammation in AML12 cells by enhancing the phosphorylation of AMPK and reducing the expression of FAS and SREBP-1c [93, 94].

Fruit and their bioactive compounds also show protective effects on AFLD *in vivo*. The consumption of lemon juice could dose-dependently alleviate alcohol-induced lipid accumulation and peroxidation in the liver [95]. Also, oral intake of blueberry juice could alleviate AFLD in C57BL/6J mice through inhibiting apoptosis by upregulating SIRT1 and downregulating the forkhead box protein O1 (FOXO1) signaling pathway [96]. Moreover, polyphenols extracted from blueberry could accelerate lipid clearance to ameliorate hepatic steatosis in C57BL/6J mice by promoting autophagy [97]. Furthermore, phenolic extracts from lychee could alleviate AFLD in C57BL/6 mice by improving hepatic steatosis, oxidative stress, and gut dysbiosis, with upregulating the Nrf2 pathway and decreasing the levels of cytochrome c, caspase-3, and the ratio of Bcl2-associated X protein (Bax) to B-cell lymphoma-2 (Bcl-2) [98, 99]. Additionally, the aqueous extract of mulberry could ameliorate AFLD in SD rats by accelerating ethanol degradation and decreasing the ratio of *Firmicutes* to *Bacteroidetes* in the gut [100]. Other fruits like Açai could alleviate alcohol-induced liver injury in Wistar rats by suppressing oxidative stress and inflammatory response by downregulating the NF-*κ*B signaling pathway [101].

**Figure 3:**
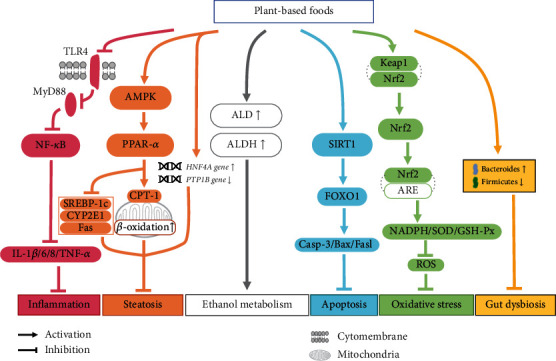
The molecular mechanisms of plant-based foods in the alleviation of AFLD. The major molecular mechanisms include the promotion of ethanol metabolism and inhibition of apoptosis, hepatic steatosis, oxidative stress, inflammation, and gut dysbiosis. First of all, plant-based foods accelerated ethanol metabolism by increasing the activities of ALD and ALDH. Also, these plants inhibited apoptosis by activating the SIRT1 pathway. Moreover, they could alleviate hepatic steatosis by activating the AMPK-PPAR-*α* pathway and regulating lipid homeostasis-related genes (like HNF4A and PTP1B). Furthermore, the antioxidative effect of such plants was related to the upregulation of the Nrf2 pathway. Besides, edible plants ameliorated inflammation by inhibiting TLR4 and NF-*κ*B pathways. Additionally, these plants attenuated gut dysbiosis by regulating the abundance of *Bacteroides* and *Firmicutes*. AMPK: adenosine 5′-monophosphate-activated protein kinase; ARE: Nrf2-antioxidant response element; CPT-1: carnitine palmitoyltransferase 1; CYP2E1: cytochrome P450 2E1; FAS: fatty acid synthase; IL-1*β*/6/8: interleukin-1*β*/6/8; NF-*κ*B: nuclear factor kappa-B; Nrf2: nuclear factor erythroid 2-related factor 2; PPAR-*α*: peroxisome proliferator-activated receptor-*α*; ROS: reactive oxygen species; SOD: superoxide dismutase; SREBP-1c: sterol regulatory element-binding transcription factor 1c; TNF-*α*: tumor necrosis factor-*α*.

In short, some fruits (such as mulberry, berries, lychee, and lemon), as well as their bioactive compounds (such as resveratrol, flavonoids, and phenolic extracts) could alleviate AFLD mainly due to the promotion of ethanol metabolism and the inhibition of liver cell apoptosis, as well as the improvement of hepatic steatosis, oxidative stress, inflammation, and gut dysbiosis (Table 1) with regulating AMPK, PPAR-*α*, Nrf2, and SIRT1 signaling pathways (Figure 3).

### 4.2. Spices and FLD

#### 4.2.1. Mechanisms of Spices and Their Bioactive Compounds on NAFLD

The *in vitro* study showed that curcumin could improve lipid accumulation by increasing the levels of CYP3A and CYP7A while decreasing the level of SREBP-1c in the primary liver cells [102]. Meanwhile, curcumin could alleviate NAFLD by decreasing the levels of lipid profiles, inhibiting O-GlcNAcylation, as well as reducing the generation of reactive oxygen species (ROS), TNF-*α*, and interferon-*γ* (IFN-*γ*) in cells [103, 104]. An *in vivo* study reported that oral intake of curcumin attenuated hepatic lipid accumulation in C57BL/6 mice by upregulating the expression of Nrf2 and farnesoid X receptor (FXR) while downregulating the expression of liver X receptor *α* (LXR*α*) [102]. Also, curcumin alleviated hepatic lipid accumulation and inflammation in C57BL/6J mice by upregulating the SIRT1-AMPK-acetyl-CoA carboxylase (ACC) signaling pathway and inhibiting the O-GlcNAcylation of NF-*κ*B [104]. Besides, recent studies also indicated that onion significantly ameliorated hepatic steatosis, ballooning, and lobular and portal inflammation in SD rats and decreased the serum levels of alanine aminotransferase (ALT), aspartate aminotransferase (AST), TG, insulin, glucose, and the level of TNF-*α* in hepatocytes [105, 106]. Collectively, spices like onion and curcumin from turmeric could prevent NAFLD through improving lipid metabolism, oxidative stress, and inflammation (Table 1) by regulating SIRT1, NF-*κ*B, and Nrf2-FXR-LXR*α* signaling pathways (Figure 2).

#### 4.2.2. Mechanisms of Spices and Their Bioactive Compounds on AFLD

Bioactive compounds from spices have been reported to fight against AFLD. Allicin, a bioactive compound from garlic, could attenuate AFLD in C57BL/6 mice by increasing the levels of glutathione (GSH), CAT, and the activity of alcohol dehydrogenase (ADH), as well as decreasing the levels of SERBP-1c, CYP2E1, TNF-*α*, and interleukins [107]. Also, allicin could alleviate alcohol-induced hepatic steatosis in C57BL/6 mice by modifying gut dysbiosis and then reducing the production of lipopolysaccharide (LPS) and further inhibiting TLR4-mediated inflammation [62]. Besides, the consumption of the ginger extract could significantly regulate lipid homeostasis in Wistar rats by increasing the expression of *hepatocyte nuclear factor 4 α* gene (*HNF4A*), while decreasing the expression of the *protein tyrosine phosphatase 1B* gene (*PTP1B*) [108, 148]. Moreover, in a mouse model of AFLD, oral gavage of curcumin could suppress the biosynthesis of fatty acids, the pathway of pentose glucuronate, and the metabolism of glyoxylate, dicarboxylate, and pyruvate [109]. To sum up, several spices (like garlic and ginger) and their bioactive compounds (like allicin) could alleviate lipid accumulation, oxidative stress, inflammation, and gut dysbiosis (Table 1), with increasing activity of ADH and the levels of antioxidant enzymes, suppressing fatty acid biosynthesis and TLR4 signaling pathway, and regulating the expressions of *HNF4A* and *PTP1B* genes (Figure 3).

### 4.3. Teas and FLD

#### 4.3.1. Mechanisms of Teas and Their Bioactive Compounds on NAFLD

Tea is one of the most popular beverages in the world and possesses hepatoprotective effects based on *in vitro* studies. The aqueous extract of green tea could moderate NAFLD in HepG2 cells by inhibiting the activity of TNF-*α* and further downregulating microRNA-34a (miR-34a) and upregulating miR-194 [110]. Besides, raw bowl tea (also called Tuocha) is a type of dark tea, and the polyphenols extracted from raw bowl tea could inhibit the proliferation of 3T3-L1 preadipocytes, suggesting its beneficial effect to alleviate NAFLD [111]. The aqueous extract of Hao Ling tea, which is a blend of green, oolong, and pu-erh tea, could inhibit the production of mitochondrial ROS in the NAFLD cell model [112]. Tea bioactive compounds also exhibit protective effects on NAFLD *in vivo*. The aqueous extract of green tea alleviated NAFLD in C57BL/6 mice by modulating the expression of miR-34a and miR-194, resulting in increasing the expression of SIRT1, PPAR-*α*, and insulin-induced gene 2 (Insig2), as well as decreasing the expression of apolipoprotein a5, 3-hydroxy-3-methyl glutaryl coenzyme A synthase (HMG-CoA synthase), and HMG-CoA reductase [110]. Also, the consumption of the green tea extract alleviated hyperlipidemia and enhanced the activity of superoxide dismutase (SOD) in Wistar rats [113]. Moreover, oral gavage of green tea polyphenols could inhibit the hepatic lipogenesis in Zucker rats by upregulating the AMPK signaling pathway, with reducing the levels of insulin, glucose, ALT, AST, TNF-*α*, and interleukin-6 (IL-6) [114]. Similarly, polyphenols from raw bowl tea could decrease the serum levels of lipid profiles and inflammatory cytokines in C57BL/6N mice [111]. More importantly, polyphenols from raw bowl tea were beneficial to alleviating NAFLD in C57BL/6N mice by regulating the abundance of gut microbiota, such as decreasing the level of *Firmicutes* and increasing the levels of *Bacteroides* and *Akkermansia* [111]. Besides, the extract of Ning Hong black tea decreased the number of hepatic lipid droplets in SD rats by upregulating the expression of PPAR-*α* and microsomal triglyceride transfer protein, as well as promoting fatty acid *β*-oxidation [115].

In short, green, black, and dark teas, as well as tea polyphenols, could alleviate NAFLD through improving liver function, regulating glucose and lipid metabolism, decreasing the levels of ROS and inflammatory cytokines, and modulating the composition of gut microbiota (Table 1), with decreasing the expression of miR-34a, increasing the expression of miR-194, and upregulating SIRT1, PPAR-*α*, and AMPK signaling pathways (Figure 2).

#### 4.3.2. Mechanisms of Teas and Their Bioactive Compounds on AFLD

It has been reported that several types of teas play a key role in alcohol metabolism [149, 150]. Green tea could accelerate ethanol metabolism in Kunming mice by increasing the activities of ADH and aldehyde dehydrogenase (ALDH), thus exerting a hepatoprotective effect [116]. Also, the consumption of green tea and its polyphenolic extract could attenuate ALFD in Wistar rats by ameliorating oxidative stress and necrosis [117, 118]. The molecular mechanisms of green tea against AFLD were due to decreasing the levels of TG, ALT, and ROS and inhibiting the expression of SREBP-1c, FAS, and CYP2E1 [117, 118]. EGCG is one of the major polyphenols in green tea, and it could alleviate ethanol-induced AFLD in Wistar rats by promoting the phosphorylation of ACC and increasing the level of CPT-1 [119, 151]. Furthermore, catechins extracted from green tea could alleviate alcohol-induced fatty changes, liver dysfunction, oxidative stress, and inflammation in Wistar rats by suppressing the NF-*κ*B signaling pathway [120].

Collectively, green tea and its bioactive compounds (like EGCG) could alleviate AFLD by improving ethanol metabolism, lipid metabolism, oxidative stress, inflammation, and necrosis (Table 1), with increasing the activities of ADH and ALDH and the phosphorylation of ACC, reducing the expression of SREBP-1c, FAS, and CYP2E1, and inhibiting the NF-*κ*B signaling pathway (Figure 3).

### 4.4. Coffee and FLD

#### 4.4.1. Mechanisms of Coffee and Its Bioactive Compounds on NAFLD

Coffee and its bioactive compounds were widely investigated [152, 153]. The *in vitro* study showed that caffeic acid could ameliorate hepatic steatosis and decrease endoplasmic reticulum stress by increasing autophagy in AML12 cells [121]. Similarly, trigonelline is one of the main components of coffee and could prevent hepatic lipid accumulation and lipotoxicity in AML 12 cells and HepG2 cells by promoting autophagy [122]. Besides, caffeine could also modulate hepatocyte steatosis by activating SIRT3 and AMPK in oleate-treated HepG2 cells [123].

The *in vivo* study showed that coffee consumption played a role in the prevention of NAFLD [124]. The coffee pulp aqueous extract improved hepatic steatosis, insulin resistance, and oxidative stress in Wistar rats by upregulating the expression of PPAR-*α*; meanwhile, the combined administration of the extract and simvastatin could additively suppress the expression of PPAR-*γ* and SREBP-1c [125]. Moreover, coffee supplementation could alleviate hepatic fat deposition and metabolic derangement in NAFLD mice by improving liver fat oxidation, intestinal cholesterol efflux, energy metabolism, and gut permeability [126]. Furthermore, trigonelline from coffee could ameliorate NAFLD in SD rats by increasing the expression of Bcl-2 protein and decreasing the expression of Bax protein in the liver [127]. Caffeine was another important bioactive compound in coffee and could suppress hepatic steatosis by activating SIRT3-AMPK-ACC and signal transducer and activator of transcription 3 (STAT3) pathways [123, 128]. Moreover, the green coffee extract showed more effectiveness in reducing hepatic TG than caffeine [129, 130]. Furthermore, decaffeinated coffee could revert to normal function the gut permeability and intestinal barrier function by increasing the expression of tight junction proteins and decreasing TLR4 expression [131]. It is worth noting that prenatal caffeine exposure of pregnant rats could increase the susceptibility of NAFLD in offspring [132].

In summary, some studies reported that coffee and its bioactive compounds (like caffeic acid, trigonelline, and caffeine) could be beneficial for NAFLD prevention through improving hepatic steatosis, insulin resistance, oxidative stress, gut permeability, and autophagy (Table 1), with regulating phosphorylation of mTOR and the expression of PPAR-*α*/*γ*, SREBP-1c, and TLR4, as well as activating the SIRT3-AMPK-ACC signaling pathway (Figure 2). But several studies showed opposite results, therefore, the hepatoprotective effects and mechanisms of coffee and caffeine still need further investigation.

#### 4.4.2. Mechanisms of Coffee and Its Bioactive Compounds on AFLD

Since caffeine possesses a strong association with liver function, several studies discussed the bioactive effect of caffeine on fat metabolism in the liver under alcohol intake [133–135]. Oral administration of caffeine could attenuate alcohol-induced hepatic cell damage, steatosis, and inflammatory response in Kunming mice by decreasing the expression of lipogenic genes and the levels of inflammatory cytokines in the serum and liver [133]. Further study showed that caffeine could even inhibit the activation of the hepatic stellate cell, thus beneficial to preventing alcohol-induced liver fibrosis in SD rats [134]. Besides, oral administration of caffeic acid to alcohol-fed Wistar rats decreased the levels of TG, total cholesterol (TC), free fatty acids, and phospholipids in circulation and the liver [135]. Collectively, caffeine and caffeic acid could ameliorate AFLD through attenuating hepatocyte damage, hepatic steatosis, and inflammatory response (Table 1), accompanied by regulating the expression of lipogenic genes and the levels of inflammatory cytokines (Figure 3).

### 4.5. Other Plants and FLD

#### 4.5.1. Mechanisms of Other Plants and Their Bioactive Compounds on NAFLD

It is well known that plenty of medicinal plants are edible foods, which have been developed into functional foods with hepatoprotective function [154, 155]. An *in vitro* study revealed that Heshouwu (*Fallopia multiflora*) could alleviate NAFLD by promoting mitochondrial *β* oxidation and attenuating lipid accumulation in L02 human liver cells [136]. Moreover, salidroside, a phenylpropanoid glycoside compound from Hongjingtian (*Rhodiola rosea*), could ameliorate NAFLD in L02 cells through alleviating steatosis and inflammation, as well as activating autophagy by downregulating the transient receptor potential melastatin2-Ca^2+^-calmodulin-stimulated protein kinase II (TRPM2-Ca^2+^-CaMKII) signaling pathway [137]. Furthermore, the treatment of silybin could alleviate NAFLD in FaO liver cells by increasing the expression of PPAR-*α*/*δ* and decreasing the expression of PPAR-*γ* [138].

The *in vivo* studies revealed that oral gavage of silymarin and silybin could ameliorate hepatic steatosis by regulating lipid metabolism and oxidative stress in C57BL/6J mice [139]. Besides, coadministration of salidroside and curcumin could alleviate high-fat diet-induced NAFLD in SD rats by activating the AMPK signaling pathway [140]. Similarly, the intake of the ethanol extract of Cassia semen (*Cassia obtusifolia*) could decrease the levels of liver enzymes, TG, TC, SOD, TNF-*α*, and interleukins in Wistar rats [141]. Furthermore, the consumption of Jishiteng (*Paederia scandens*) could reduce hepatic ROS and MDA levels by decreasing the level of heat shock cognate 71 kDa protein (HSP7C) in a chicken model of NAFLD [142].

Generally, several medicinal plants and their bioactive compounds could significantly alleviate NAFLD by regulating lipid metabolism, oxidative stress, and inflammation (Table 1). The underlying molecular mechanisms mainly included the upregulation of AMPK, Nrf2, SIRT1, and SIRT3 signaling pathways (Figure 2).

#### 4.5.2. Mechanisms of Other Plants and Their Bioactive Compounds on AFLD

According to recent experimental studies, some medicinal plants could be potential agents for the prevention of AFLD [144–146]. The *in vitro* study showed that the treatment of ginsenosides extracted from ginseng (*Panax ginseng*) could alleviate ethanol-induced hepatocyte steatosis, oxidative stress, and mitochondrial dysfunction in L02 cells by increasing the expression of PPAR-*α* and decreasing the expression of CYP2E1 [144].

The *in vivo* study showed that the intake of the aqueous extract of white flower dandelion (*Taraxacum coreanum*) ameliorated AFLD in SD rats by improving ethanol degradation, glucose metabolism, and composition of gut microbiota [100]. Besides, Zhijuzi (*Hovenia dulcis*) could alleviate hepatic steatosis and inflammation in the AFLD rat model by upregulating the expressions of PPAR-*α*, CPT-1*α*, and long-chain fatty acid CoA ligase 1 (Acsl1), as well as downregulating the expressions of myeloid differentiation factor 88 (Myd88), TNF-*α*, and C-reactive protein (CRP) [145]. Moreover, platycodin D which was extracted from Jiegeng (*Platycodon grandiflorum*) ameliorated AFLD in SD rats by inhibiting the TLR4-Myd88-NF-*κ*B signaling pathway [146]. Furthermore, *Ecklonia stolonifera* is an edible perennial brown marine alga belonging to the family *Laminariaceae* and could improve lipid metabolism in the AFLD rat model by increasing the expression of PPAR-*α* and CPT-1, accompanied by decreasing the serum levels of ALT, AST, and the hepatic level of MDA and SREBP-1c [147]

In conclusion, medicinal plants and their bioactive compounds could alleviate AFLD due to their properties of improving ethanol degradation, regulating lipid and glucose metabolism, and alleviating inflammation and gut dysbiosis (Table 1). The underlying molecular mechanisms included the upregulation of AMPK, PPAR-*α*, and ACC signaling pathways, as well as the downregulation of TLR4, Myd88, and NF-*κ*B signaling pathways (Figure 3).

## 5. Clinical Trials of Plant-Based Foods and Their Bioactive Compounds

Several clinical trials have proceeded in recent years to better verify the effects of plant-based foods, such as fruits, spices, and teas on NAFLD. A randomized cross-over clinical trial based on 61 obese children with NAFLD proved that daily consumption of 100 mL of tomato juice for 60 days could improve hepatic steatosis, insulin resistance, levels of leptin, and lipid profiles [156]. Additionally, a clinical trial found that in 55 patients, a 24-week intake of 36 g per day of dried grapes was beneficial for the prevention and management of NAFLD [157]. Moreover, the consumption of 30 to 35 g per day of dietary fiber from fruits could alleviate hepatic steatosis and improve intestinal permeability in NAFLD patients [158].

Spices and their bioactive compounds are also beneficial to FLD patients. Several double-blind randomized placebo-controlled clinical trials showed that the intake of 500 mg of green cardamom 3 times per day for 3 months could decrease the degree of fatty liver in NAFLD patients, accompanied by decreasing the levels of ALT, CRP, TNF-*α*, and IL-6, as well as increasing the serum level of SIRT1 [159]. Besides, the intake of 70 mg per day of curcumin for 8 weeks significantly reduced fatty liver in NAFLD patients, accompanied by improving the serum levels of liver enzymes, lipid profiles, glucose, and glycated hemoglobin [160]. Moreover, a clinical trial based on 55 subjects demonstrated that the supplementation of 500 mg per day of curcumin decreased the serum levels of inflammatory cytokines (like TNF-*α* and interleukins) in NAFLD patients [161]. Furthermore, a randomized, double-blind, placebo-controlled study based on 58 NAFLD patients showed that curcumin could alleviate NAFLD by regulating amino acid metabolism, tricarboxylic acid cycle, bile acid metabolism, and gut microbiota [162].

Clinical trials also indicate that oral intake of green tea shows protective effects against FLD. Oral intake of 500 mg green tea tablet containing 50 mg of standardized total polyphenols 3 times per day for 3 months effectively decreased hepatic fat accumulation, alleviated fatty liver grade, and improved liver function in 52 NAFLD patients aged 10 to 16 years [163]. Additionally, the intake of 500 mg of the green tea extract for 3 months could improve the serum levels of ALT, AST, lipid profiles, and inflammatory markers in NAFLD patients [164]. Moreover, a meta-analysis including 15 randomized clinical trials further confirmed the hepatoprotective effect of green tea and catechins against NAFLD [165].

A clinical trial with 48 NAFLD patients demonstrated that a daily dose consumption of 400 mg green coffee extract for 8 weeks could improve fasting blood glucose (mean difference (MD): −11.50; 95%CI: −19.59, −3.42), insulin resistance status (MD: −0.97; 95%CI: −1.84, −0.11), body weight (MD: −1.73; 95 % CI: −2.44, −1.01), body mass index (BMI) (MD: −0.57; 95%CI: −0.84, −0.29), and waist circumference (MD: −3.69; 95%CI: −5.85, −1.54), as well as increasing the serum level of leptin [166]. Also, the consumption of the green coffee extract increased the serum level of high-density lipoprotein cholesterol (HDL-C) (MD: 7.06; 95%CI: 0.25, 13.87) [167]. Moreover, a randomized, placebo-controlled, clinical trial with 26 NAFLD patients showed that the coadministration of 200 mg caffeine and chlorogenic acid for 12 weeks could more effectively decrease body weight than caffeine consumption alone [168]. Furthermore, a meta-analysis showed that although total caffeine intake was not related to the prevalence or progression of NAFLD, the regular intake of caffeine from daily coffee consumption could reduce the risk of hepatic fibrosis in NAFLD patients [169].

Other plant-based foods, like whole grain, also possessed a beneficial effect to alleviate NAFLD. An open-label, randomized controlled clinical trial with 112 NAFLD patients demonstrated that the consumption of whole grain for 12 weeks could alleviate hepatic steatosis and liver dysfunction [170]. Similarly, a randomized, double-blinded, parallel-armed study with 40 NAFLD patients indicated that the consumption of ancient Khorasan wheat (*Triticum turgidum*) for 3 months could reduce the levels of ALT, AST, ALP, TNF-*α*, IL-8, and IFN-*γ* [171]. Moreover, black seed (*Nigella sativa*) was a kind of edible and medicinal plant that belongs to the Ranunculaceae family, and it was very popular among Iranians [172, 173]. The consumption of 2 g per day of black seed (*Nigella sativa*) for 3 months could ameliorate hepatic steatosis in NAFLD patients [174]. Furthermore, a 3-month intake of 40 to 60 g per day of caper fruit (*Capparis spinosa*) pickle, a traditional food widely found in the western or central regions of Asia, significantly reduced the serum levels of AST and ALT and improved lipid profiles in NAFLD patients [175, 176]. In addition, a random-controlled clinical trial including 100 NAFLD patients demonstrated that a combined intake of 30 g flaxseed and 1 g hesperidin for 12 weeks could improve glucose and lipid metabolism and reduce hepatic steatosis and inflammation [177].

In short, clinical trials confirmed that the consumption of fruits (such as tomato and grapes), spices (like green cardamom), teas (mainly green tea), coffee, and other plant-based foods (like whole grain, black seed, and caper), as well as their bioactive compounds (such as dietary fiber and curcumin), could alleviate NAFLD in human.

## 6. Safety and Application Issues of Plant-Based Foods and Their Bioactive Compounds in Patients

The plant-based, high-fiber, and low-fat diet has been recognized as a healthy lifestyle and recommended to manage FLD for a long time. It is very necessary to evaluate the safety and application issues of plant-based foods and their bioactive compounds.

### 6.1. Safety and Application Issue of Fruits and Their Bioactive Compounds

The pharmacokinetic study revealed that after ingesting 500 mg of resveratrol for 24 hours, there were no adverse reactions for all healthy volunteers, and the maximum concentration and the area under the curve of resveratrol were lower when compared with its metabolites in plasma [178]. Besides, a double-blind, randomized, placebo-controlled trial based on 32 overweight senior citizens (mean age: 73 ± 7 years) showed that the daily consumption of 300 or 1,000 mg resveratrol for 90 days did not adversely affect blood chemistries [179]. It was worth noting that a clinical trial showed that the daily intake of 3,000 mg resveratrol in overweight or obese men with NAFLD for 8 weeks did not improve any features of NAFLD but increased the levels of liver enzymes [180].

Berries are recognized as healthy foods and rich in anthocyanins [181]. The *in vivo* study revealed that the acute oral median lethal dose of the mixture extract from common edible berries was higher than 5 g/kg in SD rats, which means that the mixture extract of berries was a relatively safe food [182]. To further assess the safety of anthocyanin-rich fruit consumption in human, a pilot study tested the biochemical changes in blood after ingesting sweet cherries at the dose of three cups per day for 4 weeks, and it turned out that 37 overweight senior citizens were well tolerated to the intervention [183]. Moreover, 8-week treatment with 200 mg of acylated anthocyanin from purple sweet potato twice a day in NAFLD patients could reduce the levels of liver enzymes, especially *γ*-glutamyl transpeptidase [184]. Furthermore, a CONSORT-compliant, randomized, double-blind, placebo-controlled pilot trial showed that the consumption of anthocyanin derived from bilberry and black currant at a dose of 320 mg per day for 12 weeks attenuated clinical symptoms in 74 NAFLD patients without side effect reported [185].

Z-isomers of lycopene are abundant in processed tomato products (like tomato oleoresin) with high bioavailability, and the median lethal dose of tomato oleoresin was more than 5,000 mg/kg (which was equal to 361 mg/kg of (Z)-lycopene) via a single-dose oral test in Wistar rats [186]. Further, in a repeated-dose toxicity test, after oral administrating tomato oleoresin at a dose of 4,500 mg/kg per day (which was equal to 325 mg/kg/day of (Z)-lycopene) for 4 weeks, the Wistar rats showed no adverse changes [186]. Only a few studies tried to use lycopene as a potential treatment against FLD with no adverse effects reported so that more evidence is needed on the topic [156].

The intake of fructose is recognized as a risk factor for NAFLD, and some types of fruit are fructose-rich foods [187]. A case report of a 33-year-old male with insulinoma and hypoglycemia for 4 years showed that overeating of fruits, especially the fruits rich in fructose, might exacerbate histological changes of NAFLD [188]. Therefore, the dietary recommendations of fruit intake for the fatty liver population need to be considered thoroughly and carefully.

### 6.2. Safety and Application Issue of Spices and Their Bioactive Compounds

A randomized controlled trial reported that NAFLD patients who received curcumin at a dose of 1,000 mg per day for 8 weeks revealed a reduction of the body mass index, waist circumference, and the levels of liver enzymes with good tolerance [189]. Moreover, an 8-week single-arm trial on 36 NAFLD patients showed that the consumption of the curcumin-phospholipid complex at a dose of 1,500 mg per day could ameliorate NAFLD severity and none of the severe adverse events were reported [190]. Besides, a clinical trial based on 98 NAFLD patients showed that the intake of 800 mg garlic for 15 weeks could improve hepatic steatosis compared with the placebo group (RR: 5.6; 95%CI: 2.17, 14.5) with no serious adverse effects [191]. Allicin is a common phytochemical from garlic, and a previous high-quality article summarized the safety assessment of allicin application and emphasized that the high-dose consumption of allicin (30 to 59 mg) could cause stomach irritation, especially when people were in a fasting state or ingested too fast [192].

### 6.3. Safety and Application Issue of Teas and Their Bioactive Compounds

Tea polyphenol and its major component EGCG are commonly recognized as healthy and safe foods [193, 194]. A double-blind randomized clinical trial on 45 NAFLD patients showed that daily received 550 mg of green tea tablets for 3 months significantly improved the body mass index, level of AST, and fasting blood sugar [195]. Even though green tea was famous for its therapeutic effects on NAFLD, it is still needed to be cautious about its side effect [196]. A 4-week clinical trial on 40 healthy participants found out that the intake of EGCG at a dose of 400 or 800 mg once per day led to a few mild adverse events, such as excess gas, upset stomach, nausea, heartburn, stomach ache, abdominal pain, dizziness, headache, and muscle pain [197]. Meanwhile, the consumption of EGCG at 800 mg per day could significantly increase the systemic availability of free EGCG by more than 60% [197].

### 6.4. Safety and Application Issue of Coffee and Its Bioactive Compounds

The coffee fruit contains a series of bioactive compounds, such as phenolic acids, methylxanthines, chlorogenic acids, and caffeine, which can be used as active ingredients of functional foods or drugs [198]. In the acute test, the oral administration of the hydroethanolic extract from green coffee fruit at a dose of 1,000 mg/kg for 14 days did not show adverse effects on mice, and the oral median lethal dose of the extract was 5,000 mg/kg per day [198]. Besides, the increase in workout time in daily life has been recognized as a healthy lifestyle and beneficial to FLD management, and caffeine is a common food additive used in preworkout supplements [199]. A double-blinded, placebo-controlled study with 17 males reported that the daily consumption of caffeine 20 min before exercise at a dose of 300 mg for 28 days showed no adverse changes on the biomarkers of renal and liver functions [199]. Moreover, the oral administration of green coffee oil rich in cafestol and kahweol did not lead to adverse effects in rats based on the acute toxicity test and the subacute toxicity test [200]. Furthermore, an *in vivo* study reported that spent coffee grounds did not show mycotoxins and toxicity based on the pilot repeated intake study with Wistar rats and could be a sustainable and safe ingredient for preventing hepatic steatosis [201].

### 6.5. Safety and Application Issue of Other Plants and Their Bioactive Compounds

According to the results of a multicenter phase II clinical trial, 78 patients with nonalcoholic steatohepatitis received silymarin at a dose of 420 or 700 mg for 48 weeks in which we did not observe significant adverse events, indicating that a higher than customary doses of silymarin treatment were safe and well tolerated [202]. Besides, salidroside, a bioactive component extracted from Hongjingtian (*Rhodiola rosea*), was evaluated as genotoxicity free via the reverse mutation assay, chromosomal aberration assay, and mouse micronucleus assay [203]. In addition, ginsenosides are bioactive compounds from ginseng (*Panax ginseng*) with low absorption and bioavailability, which made the clinical application of ginsenosides became very difficult; therefore, further studies are needed to develop the advanced delivery systems to enhance the clinical usefulness of ginsenosides [77].

To sum up, although the current mainstream view and study results indicate that increasing the proportion of plant-based food intake in the dietary diet is overall beneficial to the prevention and management of FLD, there are still some issues that remain to be resolved in the practical application. It is pleasant to notice that more and more clinical trials can further explore the new evidence of the safety and efficacy of plant-based foods and their bioactive compounds on FLD [204, 205].

## 7. Conclusions

In conclusion, millions of people suffered from FLD (including NAFLD and AFLD) and the numbers are still climbing year by year, despite the vigorous promotion of more exercise, weight control, and alcohol abstinence. Epidemiological evidence suggests that a healthy dietary pattern with increasing the intake of several plant-based foods could lower the risk of FLD. Further experimental studies discovered that the mechanisms of plant-based foods and their bioactive compounds against FLD included the improvement of hepatic steatosis, oxidative stress, inflammation, gut dysbiosis, apoptosis, autophagy, and ethanol metabolism. Moreover, the upregulation of AMPK, SIRT1, Nrf2, and PPAR-*α* signaling pathways and the downregulation of PPAR-*γ*, NF-*κ*B, mTOR, Akt, and TLR4 signaling pathways played a crucial role in the remission of FLD. More importantly, since the absence of standardized therapies for FLD, a hypocaloric diet accompanied by physical activity is recommended to manage fatty liver as reported in the international guidelines. Several clinical trials confirmed the protective effects of plant-based foods against FLD. In this context, increased consumption of plant-based foods in daily life can be efficient to treat FLD patients. Furthermore, the practical application of plant-based foods and their bioactive compounds in FLD patients is well tolerated, but the safe and effective dose needs to be further confirmed. In the future, more plant-based foods need to be investigated as potentially functional foods on FLD prevention and their main bioactive compounds need to be separated and identified.

## Figures and Tables

**Table 1:** The protective effects and molecular mechanisms of plant-based foods against NAFLD and AFLD in experimental studies.

Plant-based foods	Bioactive component	Study type	Models	Doses	Main effects	Molecular mechanisms	Ref
Fruits—NAFLD							
Grape	Resveratrol	*In vitro*	HepG2 cells	12.5, 25, 50, 100 *μ*M	Inhibiting lipogenesis and proliferation	Not mentioned	[79]
Grape (red wine)	Resveratrol	*In vitro*, *in vivo*	HepG2 cells SD rats	40 *μ*M 100 mg/kg	Alleviating lipid accumulation and oxidative stress	Activating the PKA-AMPK-PPAR-*α* pathway	[80]
	Resveratrol	*In vitro*, *in vivo*	HepG2 cells, C57BL/6 mice	20 *μ*M for cells 0.4 % in the chow	Reducing the expression of FAS and SREBP-1c genes	Inhibiting methylation of Nrf2 promoter	[81]
	Rutin	*In vitro*, *in vivo*	HepG2 cells, RAW 246.7 cells, C57BL/6 mice	10, 20, 40 *μ*M 200 mg/kg	Inhibiting lipogenesis, oxidative injuries, and autophagy	Activating the PPAR-*α* pathway	[82]
Sweet cherry	Anthocyanins	*In vitro*	HepG2 cells LO2 cells	100, 200, 300 *μ*g/mL	Inducing autophagy	Activating AMPK and inhibiting mTOR and Akt pathways	[83]
Grape	Polymerized anthocyanin	*In vivo*	C57BL/6J mice	400 mg/kg	Improving liver function, dyslipidemia, and hepatic steatosis	Activating Nrf2 and SIRT1 and inhibiting PPAR-*γ* pathways	[84]
Tomato		*In vivo*	SD rats	Freely drink	Alleviating gut dysbiosis	Increasing *Lactobacillus* abundance and diminishing the acetate/propionate ratio	[85]
	Lycopene	*In vivo*	SD rats Wistar rats	5, 10, 20 mg/kg	Alleviating liver injury, lipid accumulation, fat infiltration, and oxidative stress	Reverting activities of SOD, GSH, and CAT and decreasing TNF-*α* and CYP2E1 levels	[86–88]
Acerola cherry	Polysaccharide	*In vivo*	C57BL/6 mice	200, 400, 800 mg/kg/day	Improving mitochondrial function, lipogenesis, oxidative stress, and inflammation	Inhibiting SREBP-1c and activating PGC-1*α* and Nrf2 pathways	[89]
Mulberry		*In vivo*	SD rats	100, 200 mg/kg	Alleviating liver damage, dyslipidemia, and oxidative stress	Improving mitochondrial function	[90]
Açai		*In vivo*	Fischer rats	20 g/kg	Alleviating steatosis and inflammation	Reducing liver enzymes and increasing GSH/GSSG	[91]
Fruits—AFLD							
	Resveratrol	*In vitro*	HepG2 cell	5, 15, 45, 135 *μ*M	Reducing lipid accumulation	Activating the AMPK-lipin1 pathway	[92]
	Myricitrin Myricetin	*In vitro*	AML12 cells	5, 10, 20, 40 *μ*M 60*μ*M	Alleviating steatosis, oxidative stress, and inflammation	Activating the AMPK pathway	[93, 94]
Lemon		*In vivo*	C57BL/6 mice	10 mL/kg	Alleviating lipid accumulation and lipid peroxidation	Decreasing the levels of SOD and CAT	[95]
Blueberry	Polyphenols	*In vivo*	C57BL/6J mice	1.5 mL/100 g 100, 200 mg/kg/day	Inhibiting apoptosis and promoting autophagy	Activating SIRT1 and inhibiting FOXO1 pathways	[96, 97]
Lychee	Phenols	*In vivo*	C57BL/6 mice	150, 300 mg/kg	Alleviating steatosis, oxidative stress, and gut dysbiosis	Activating the Nrf2 pathway, decreasing cytochrome c, caspase-3 activities, and Bax/Bcl-2 ratio	[98, 99]
Mulberry		*In vivo*	SD rats	0.3 g/kg	Improving steatosis, gut dysbiosis, and glucose metabolism	Accelerating ethanol degradation, decreasing ratio of Firmicutes to Bacteroidetes	[100]
Açai		*In vivo*	Wistar rats	1 mL/100 g	Alleviating oxidative stress and inflammation	Inhibiting the NF-*κ*B pathway	[101]
Spices—NAFLD							
	Curcumin	*In vitro* *In vivo*	Primary liver cells C57BL/6 mice	10 *μ*M 50, 100 mg/kg	Regulating bile acids and exogenous xenobiotic metabolism	Increasing Nrf2 and FXR and decreasing LXR*α* levels	[102]
	Curcumin	*In vitro* *In vivo*	PBM cells C57BL/6J mice	30 *μ*M for cells 2 g/kg in the chow	Preventing intrahepatic CD4+ cell accumulation, oxidative stress, and inflammation	Inhibiting the production of ROS, TNF-*α*, and IFN-*γ*	[103]
	Curcumin	*In vitro* *In vivo*	AML12 cells C57BL/6J mice	0.3, 3 *μ*M 100 mg/kg	Alleviating lipid accumulation, oxidative stress, and inflammation	Inhibiting O-GlcNAcylation of NF-*κ*B and upregulating the SIRT1-AMPK-ACC pathway	[104]
Onion		*In vivo*	SD rats	7% *w*/*w*	Alleviating steatosis, ballooning, and lobular and portal inflammation	Decreasing levels of TNF-*α*, ALT, AST, TG, insulin, and glucose	[105, 106]
Spices—AFLD							
Garlic	Allicin	*In vivo*	C57BL/6 mice	5, 20 mg/kg/day	Alleviating oxidative stress and inflammation	Reducing the levels of SERBP-1c, CYP2E1, TNF-*α*, IL-1*β*, and IL-6, increasing the levels of GSH, CAT, and the activity of ADH	[107]
Garlic	Allicin	*In vivo*	C57BL/6 mice	5, 20 mg/kg/day	Alleviating steatosis, inflammation, and gut dysbiosis	Inhibiting the LPS-TLR4 pathway	[62]
Ginger		*In vivo*	Wistar rats	50 mg/kg	Alleviating lipid accumulation and liver enzyme changes	Increasing the expression of HNF4A and decreasing the expression of the PTP1B gene	[108]
	Curcumin	*In vivo*	Kunming mice	60 mg/kg	Suppressing fatty acid biosynthesis and pentose glucuronate pathway	Inhibiting the metabolisms of glyoxylate, dicarboxylate, and pyruvate	[109]
Tea—NAFLD							
Green tea	Catechins	*In vitro* *In vivo*	HepG2 cells C57BL/6 mice	2 *μ*M and 0.19% 500 mg/kg	Alleviating lipid accumulation, increasing gene expression related to catabolism of TG and fatty acid	Downregulating miR-34a and upregulating miR-194	[110]
Raw bowl dark tea	Polyphenol	*In vitro* *In vivo*	3T3-L1 preadipocytes C57BL/6N mice	200 *μ*g/mL 50, 100 mg/kg/day	Alleviating lipid accumulation, oxidative stress, and inflammation and improving the intestinal environment	Increasing the levels of occludin, ZO-1, *Bacteroides*, and *Akkermansia* and reducing the level of *Firmicutes*	[111]
Hao Ling tea	Polyphenol Caffeine	*In vitro* *In vivo*	Primary liver cells Wistar rats	100, 250, 500 *μ*g/mL 10% in the drink	Alleviating hepatic steatosis and oxidative stress	Inhibiting the production of mitochondrial ROS	[112]
Green tea	Phenols Flavonoids	*In vivo*	Wistar rats	300 mg/kg	Improving hyperlipidemia and oxidative stress	Increasing the activity of SOD	[113]
Green tea	Polyphenol	*In vivo*	Zucker rats	200 mg/kg	Decreasing lipogenesis, levels of insulin, glucose, liver enzymes, TNF-*α*, and IL-6	Upregulating the AMPK pathway	[114]
Ning Hong black tea		*In vivo*	SD rats	2% in the chow	Decreasing the body fat ratio and the number of lipid droplets in the liver	Upregulating expression of PPAR-*α* and MTP, promoting fatty acid *β*-oxidation and VLDL synthesis	[115]
Tea—AFLD							
Green tea		*In vivo*	Kunming mice	10 mL/kg	Improving ethanol metabolism and liver function	Increasing the activities of ADH and ALDH	[116]
Green tea	Catechins Caffeine	*In vivo*	Wistar rats	20 mL/kg/day	Alleviating lipogenesis and oxidative injury	Reducing levels of SREBP-1c, FAS, CYP2E1, and NADPH oxidase p47phox protein	[117]
Green tea	EGCG	*In vivo*	Wistar rats	300 mg/kg/day	Alleviating oxidative stress and necrosis	Decreasing levels of TNF-*α* and 4-hydroxynonenal	[118]
Green tea	EGCG	*In vivo*	Wistar rats	3 g/L	Improving fatty liver and the levels of ALT and AST	Increasing the phosphorylation of ACC and the level of CPT-1	[119]
Green tea	Catechin	*In vivo*	Wistar rats	50 mg/kg/day	Alleviating fatty changes, liver dysfunction, and oxidative stress	Inhibiting the NF-*κ*B pathway	[120]
Coffee—NAFLD							
	Caffeic acid	*In vitro* *In vivo*	AML 12 C57BL/6 mice	12.5, 25, 50, 100, 200 *μ*M 50 mg/kg/day	Alleviating steatosis endoplasmic reticulum stress and increasing autophagy	Activating the Akt pathway	[121]
	Trigonelline	*In vitro* *In vivo*	AML 12 HepG2 cells C57BL/6J mice	50, 200 *μ*M 50 mg/kg	Alleviating steatosis and lipotoxicity and promoting autophagy	Deceasing the phosphorylation of mTOR and the expression of PPAR-*γ*, SREBP-1, perilipin, and CD36	[122]
Coffee	Caffeine	*In vitro* *In vivo*	HepG2 cells C57BL/6 mice	2 mM 10, 20 mg/kg	Alleviating steatosis	Activating the SIRT3-AMPK-ACC pathway	[123]
Coffee	Caffeine	*In vivo*	Tsumura Suzuki nonobese mice	1 g/L, freely drink	Inhibiting pancreatic-*β* cell damage and nonalcoholic steatohepatitis	Not mentioned	[124]
Coffee		*In vivo*	Wistar rats	1000 mg/kg	Alleviating steatosis, insulin resistance, and oxidative stress	Upregulating the expression of PPAR-*α*	[125]
Coffee		*In vivo*	C57BL/6J mice	4:1, *v*/*w*, freely drink	Improving liver fat oxidation, intestinal cholesterol efflux, energy metabolism, and gut permeability	Upregulating the expression of PPAR-*α*, acyl-CoA oxidase-1, ABCA1, ABCG1, zonulin-1, claudin, and peptide YY, as well as increasing the abundance of *Alcaligenaceae*	[126]
	Trigonelline	*In vivo*	SD rats	40 mg/kg/day	Alleviating steatosis and the damage degree of the liver	Increasing the level of SOD and the expression of Bcl-2	[127]
Coffee	Caffeine	*In vivo*	C57BL/6J mice	0.5 mg/mL, freely drink	Alleviating steatosis	Activating STAT3 in the liver and increasing IL-6 in circulation	[128]
Coffee	Caffeine	*In vivo*	SD rats	8 g/180 mL in drinking water, 0.18 g/kg in diet	Alleviating steatosis	Decreasing the phosphorylation of mTOR and increasing the level of nuclear lipin1	[129, 130]
Coffee		*In vivo*	Wistar rats C57BL/6J mice	6 g/kg	Improving the gut permeability and intestinal barrier function	Increasing the expression of occludin and ZO-1, decreasing the expression of TLR4	[131]
Coffee	Caffeine	*In vivo*	Wistar rats	30, 60, 120 mg/kg/day	Increasing the susceptibility of NAFLD in offspring	Inhibiting the expression of SIRT1	[132]
Coffee—AFLD							
Coffee	Caffeine	*In vivo*	Kunming mice	5, 10, 20 mg/kg	Alleviating hepatic cell damage, steatosis, and inflammatory response	Decreasing the expression of SERBP-1c, Fas, ACC, SCD 1, and the levels of TNF-*α*, IL-1*β*/6, IFN-*γ*, and MCP-1	[133]
Coffee	Caffeine	*In vivo*	SD rats	5, 10, and 20 mg/kg/day	Inhibiting the activation of hepatic stellate cell	Inhibiting the PKA pathway	[134]
Coffee	Caffeic acid	*In vivo*	Wistar rats	12 mg/kg/day	Decreasing the levels of TG, TC, free fatty acids, and phospholipids in the circulation and liver		[135]
Other plants—NAFLD							
Heshouwu (*Fallopia multiflora*)	Stilbenes anthraquinones	*In vitro* *In vivo*	L02 cell Wistar rats	3.75, 7.5, 15, 30, 60 *μ*g/mL 70, 140, 280 mg/kg	Improving mitochondrial *β* oxidation and dyslipidemia	Increasing the expression of CPT-1*α*	[136]
Hongjingtian (*Rhodiola rosea*)	Salidroside	*In vitro*	L02 cell	75, 150, 300 *μ*g/mL	Alleviating steatosis, inflammation, and activating autophagy	Inhibiting the TRPM2-Ca2+-CaMKII pathway	[137]
	Silybin	*In vitro*	FaO cells	50 *μ*M	Alleviating fat accumulation and mitochondrial damage	Increasing the expression of PPAR-*α*/*δ* and decreasing the expression of PPAR-*γ*	[138]
	Silybin	*In vivo*	C57BL/6J mice	50, 100 mg/kg/day	Regulating lipid metabolism and global metabolic pathways	Modulating the metabolisms of lipid, polyol, amino acid, urea cycle, and TCA cycle	[139]
	Curcumin and salidroside	*In vivo*	SD rats	21.76 mg/kg/d and 5.77 mg/kg/d	Alleviating insulin resistance and lipid peroxidation	Activating the AMPK pathway	[140]
Cassia (*Cassia obtusifolia*)		*In vivo*	Wistar rats	0.5, 1, 2 g/kg	Alleviating histopathological changes, dyslipidemia, and lipid peroxidation in the liver	Increasing the activities of SOD and GSH	[141]
Jishiteng (*Paederia scandens*)		*In vivo*	Ross 305 chicks	2 mg/kg	Alleviating oxidative stress	Decreasing the level of HSP7C	[142]
Heshouwu (*Fallopia multiflora*)		*In vivo*	Zebrafish	0.5, 1 mg/mL 0.25, 0.5 *μ*g/mL	Reducing lipogenesis and insulin resistance	Activating the PI3K-Akt2 -AMPK-PPAR-*α* pathway	[143]
Other plants—AFLD							
Ginseng (*Panax ginseng*)	Ginsenosides	*In vitro*	L02 cells	25, 50, 100 *μ*g/mL	Alleviating steatosis, oxidative stress, and mitochondrial dysfunction	Increasing the expression of PPAR-*α* and decreasing the expression of CYP2E1	[144]
White flower dandelion (*Taraxacum coreanum*)		*In vivo*	SD rats	1 g/kg/day	Improving body composition, glucose metabolism, ethanol degradation, and gut dysbiosis	Decreasing the ratio of *Firmicutes* to *Bacteroidetes*	[100]
Zhijuzi (*Hovenia dulcis*)		*In vivo*	SD rats	300, 500 mg/kg	Alleviating steatosis and inflammation	Increasing PPAR-*α*, PPAR-*γ*C1*α*, CPT-1*α*, and Acsl1 gene expression, decreasing Myd88, TNF-*α*, and CRP gene expression	[145]
*Platycodon grandiflorum*	Platycodin D	*In vivo*	SD rats	10, 20, 30 mg/kg/day	Inhibiting inflammation and endotoxic process	Inhibiting the TLR4-MyD88-NF-*κ*B pathway	[146]
*Ecklonia stolonifera*	Phlorotannins	*In vivo*	SD rats	50, 100, 200 mg/kg/day	Improving liver function and lipid profiles	Increasing the expression of PPAR-*α* and CPT-1 and decreasing the expression of SREBP-1c	[147]

ABCA1: ATP-binding cassette subfamily A1; ABCG1: ATP-binding cassette subfamily G1; ACC: acetyl-CoA carboxylase; ADH: alcohol dehydrogenase; Akt: protein kinase B; ALT: alanine aminotransferase; AMPK: adenosine 5′-monophosphate-activated protein kinase; ALDH: aldehyde dehydrogenase; ALP: alkaline phosphatase; AST: aspartate aminotransferase; Bax: Bcl2-associated X protein; Bcl-2: B-cell lymphoma-2; CAT: catalase; CPT-1: carnitine palmitoyltransferase 1; CYP2E1: cytochrome P450 2E1; EGCG: epigallocatechin gallate; FAS: fatty acid synthase; FOXO1: forkhead box protein O1; FXR: farnesoid X receptor; GSH: glutathione; GSH-Px: glutathione peroxidase; GSSG: oxidized glutathione; HNF4A: hepatocyte nuclear factor 4 *α*; IL-1*β*/6/8: interleukin-1*β*/6/8; IFN-*γ*: interferon-*γ*; LPS: lipopolysaccharide; LXR*α*: liver X receptor *α*; MCP-1: monocyte chemoattractant protein 1; MDA: malondialdehyde; miR-34a/194: microRNA-34a/194; mTOR: mammalian target rapamycin; MTP: microsomal triglyceride transfer protein; NADPH: nicotinamide adenine dinucleotide phosphate; NF-*κ*B: nuclear factor kappa-B; Nrf2: nuclear factor erythroid 2-related factor 2; PBM cells: peripheral blood mononuclear cells; PGC-1*α*: peroxisomal proliferator-activated receptor-gamma coactivator-1*α*; PKA: protein kinase A; PPAR-*α*/*γ*: peroxisome proliferator-activated receptor-*α*/*γ*; PTP1B: protein tyrosine phosphatase 1B; ROS: reactive oxygen species; SCD 1: stearoyl-CoA desaturase 1; SD rats: Sprague-Dawley rats; SIRT1: sirtuin 1; SOD: superoxide dismutase; SREBP-1c: sterol regulatory element-binding transcription factor 1c; STAT3: signal transducer and activator of transcription 3; TG: triglyceride; TLR 4: toll-like receptor 4; TNF-*α*: tumor necrosis factor-*α*; VLDL: very low-density lipoprotein; ZO-1: zonula occludens-1.
